# Insulin exerts anti-inflammatory effects through reduction of IKK/IκB/NF-κB pathway activation in septic rats

**DOI:** 10.1186/cc11755

**Published:** 2012-11-14

**Authors:** F Mittestainer, B de Melo Carvalho, K Lima Calisto, D Guadagnini, M Jose Abdalla Saad

**Affiliations:** 1 University of Campinas, Brazil

## Background

During the onset of sepsis, the inflammatory system becomes hyperactive, leading to an overproduction of proinflammatory mediators, which contribute to septic shock, multiple organ failure, and death. Animal studies indicate that insulin may have direct anti-inflammatory effects, independent of its effect on hyperglycemia. However, the mechanism by which insulin reduces inflammation in the absence of hyperglycemia is unknown. The aim of the present study was to investigate of the molecular mechanisms of action and response of insulin administration in relation to its anti-inflammatory effect in septic animals. We analyzed the modulation of IKK/IκB/NF-κB pathways and insulin signaling pathway (IR, IRS-1 and Akt) and the expression of NF-κB p65 DNA binding in liver, muscle and adipose tissue in septic animals.

## Methods

For the experiment, diffuse sepsis was induced by cecal ligation and puncture surgery in male adult Wistar rats. The septic animals were randomly divided into four different groups. Three of them were submitted to insulin injection in the portal vein and were stimulated for 1, 3 and 5 minutes, and the other group (control) was not stimulated (saline). After the stimulation time, insulin sensitive tissues were extracted and the expression and phosphorylation of the proteins were analyzed through western blot. NF-κB p65 activation was determined in nuclear extracts from muscle, liver and adipose tissue from septic rats by ELISA.

## Results

In a time-course experiment we observed an increase in insulin-induced phosphorylation of IR-β, IRS1 tyrosine phosphorylation, and Akt serine phosphorylation that reached a peak at 3 minutes post injection in muscle, liver and adipose tissue in septic animals. In parallel, there was a reduction in the phosphorylation of IKK in the time 1 and 3 minutes after stimulation by insulin returned to basal levels at 5 minutes. There was also a reduction in the phosphorylation of IκB in the time 3 and 5 minutes after insulin injection. The DNA binding of NF-κB p65 in the cell nucleus showed a reduction in expression levels of NF-κB at all times investigated, in accordance with the reduction of IKK and IκB phosphorylation after insulin injection in liver, muscle and adipose tissue of septic animals.

## Conclusion

In summary, we report evidence indicating that the acute infusion of insulin activated the PI3K-Akt pathway and reduced IKK/IκB/NF-κB indicate an important molecular mechanism to the anti-inflammatory effect of the hormone in liver, muscle and adipose tissue of septic animals. This helps explain a correlation between activation of insulin signaling and its effect on the inflammatory pathway.

